# MicroRNA-302b mitigates renal fibrosis via inhibiting TGF-β/Smad pathway activation

**DOI:** 10.1590/1414-431X20209206

**Published:** 2021-01-25

**Authors:** Mengkui Sun, Wei Zhou, Fei Yao, Jianming Song, Yanan Xu, Zhimei Deng, Hongwang Diao, Shoulin Li

**Affiliations:** 1 Department of Urology, Shenzhen Children's Hospital, Shenzhen, Guangdong Province, China; 2 Laboratory of Pelvic Floor Muscle Function, Shenzhen Children's Hospital, Shenzhen, Guangdong Province, China; 3 Department of Pathology, Shenzhen Children's Hospital, Shenzhen, Guangdong Province, China

**Keywords:** MicroRNA-302b, Renal fibrosis, Epithelial mesenchymal transition, TGF-β receptor 2, Unilateral ureteral obstruction

## Abstract

Renal fibrosis is one of the most significant pathological changes after ureteral obstruction. Transforming growth factor-β (TGF-β) signaling pathway plays essential roles in kidney fibrosis regulation. The aims of the present study were to investigate effects of microRNA-302b (miR-302b) on renal fibrosis, and interaction between miR-302b and TGF-β signaling pathway in murine unilateral ureteral obstruction (UUO) model. Microarray dataset GSE42716 was downloaded by retrieving Gene Expression Omnibus database. In accordance with bioinformatics analysis results, miR-302b was significantly down-regulated in UUO mouse kidney tissue and TGF-β1-treated HK-2 cells. Masson's trichrome staining showed that miR-302b mimics decreased renal fibrosis induced by UUO. The increased mRNA expression of collagen I and α-smooth muscle actin (α-SMA) and decreased expression of E-cadherin were reversed by miR-302b mimics. In addition, miR-302b up-regulation also inhibited TGF-β1-induced epithelial mesenchymal transition (EMT) of HK-2 cells by restoring E-cadherin expression and decreasing α-SMA expression. miR-302b mimics suppressed both luciferase activity and protein expression of TGF-βR2. However, miR-302b inhibitor increased TGF-βR2 luciferase activity and protein expression. Meanwhile, miR-302b mimics inhibited TGF-βR2 mRNA expression and decreased Smad2 and Smad3 phosphorylation *in vivo* and *in vitro*. Furthermore, over-expression of TGF-βR2 restored the miR-302b-induced decrease of collagen I and α-SMA expression. In conclusion, this study demonstrated that miR-302b attenuated renal fibrosis by targeting TGF-βR2 to suppress TGF-β/Smad signaling activation. Our findings showed that elevating renal miR-302b levels may be a novel therapeutic strategy for preventing renal fibrosis.

## Introduction

Obstructive nephropathy leads to almost 30-50% of all end-stage renal disease cases in infants and children (1). Ureteropelvic junction obstruction is the most common etiology of congenital urinary tract obstruction. During ureteral obstruction, increased synthesis of angiotensin II, cytokines, and growth factors are identified as causative factors that result in renal interstitial fibrosis characterized by infiltration of inflammatory cells, accumulation of activated myofibroblasts, extracellular matrix proteins deposition, and tubular cell damage (2). Renal interstitial fibrosis impedes the normal function of tubules and glomerulus. Unfortunately, there is still no effective treatment to specifically inhibit or reverse renal interstitial fibrosis before or after surgical intervention. However, it has been demonstrated that transforming growth factor-β (TGF-β)/α-smooth muscle actin (Smad) signaling plays a vital role in renal fibrosis pathogenesis (3). Therefore, intervention of the TGF-β/Smad signaling pathway may benefit renal fibrosis prevention.

MicroRNAs, approximately 20∼25 nucleotides long, are small endogenous single-stranded non-coding RNAs that exhibit negative characters after gene transcription through binding to the 3′-untranslated region of target gene mRNA (4). Hence, aberrant expression of miRNAs participates in numerous pathologic processes including cancer, diabetes, and myocardial disease (5–7). In addition, accumulating reports have demonstrated that miRNAs play important roles in the progression of renal fibrosis. In a mouse diabetic nephropathy renal fibrosis model, miR-377 is up-regulated and results in increased expression of fibronectin protein, which is a fundamental matrix protein in renal fibrosis (8). Another study demonstrates that miR-200a and miR-141, two members of the miR-200 family, can enhance E-cadherin protein expression by targeting zinc finger E-box-binding homeobox (ZEB) 1 and ZEB2. Hence, miR-200a and miR-141 can decrease TGF-β1-induced tubular epithelial-to-mesenchymal transition (EMT), which plays crucial roles in the progression of renal fibrosis (9). Furthermore, miR-433 is proven to decrease Azin1 expression which, in turn, activates TGF-β/Smad3 pathway and the fibrotic response (10). These studies indicate the possibility of renal fibrosis prevention by miRNAs regulation.

Unilateral ureteral obstruction (UUO) is a widely applied experimental animal model for renal interstitial fibrosis (11). The aims of the present study were to investigate the effects of microRNA-302b (miR-302b) on renal fibrosis and to determine the interaction between miR-302b and TGF-β signaling pathway in the murine UUO model.

## Material and Methods

### Reagents

miR-302b mimics, miR-302b inhibitor, and corresponding negative control oligonucleotides were purchased from GenePharma (China). Adeno-associated virus encoding miR-302 mimic or negative control were obtained from Oobio (China). Human renal proximal tubular epithelial (HK-2) cells were obtained from the American Type Culture Collection (ATCC, USA). DMEM/F12, fetal bovine serum (FBS), penicillin, streptomycin, and TGF-β1 were from Invitrogen (USA).

### Data resources

MicroRNA expression profile GSE42716, which includes 4 UUO samples and 4 normal samples, was obtained from the Gene Expression Omnibus (GEO) database (NCBI, USA).

### Analysis of differential expression miRNAs

GEO2R (http://www.ncbi.nlm.nih.gov/geo/geo2r/), which is based on the Limma R and GEOquery packages, is a convenient online tool that performs comparisons on GEO datasets. Differential expression analysis of mi-RNAs between control group and UUO group was carried out using the GEO2R program. The cut-off criteria were set as P-value <0.05 and |log fold change| >2 for identifying differential expression miRNAs.

### Prediction of target genes

The potential target genes of miR-302b were predicted by TargetScan (http://www.target-scan.org/mmu_71/).

### Animal model of UUO

All experiments were conducted in 6∼8-week-old ICR male mice, weighing 23∼26 g, obtained from Guangdong Medical Laboratory Animal Center (China). Animals were kept with free access to rodent food and clean water on a normal circadian rhythm. All procedures were approved by the Institutional Animal Care and Use Committee of Shantou University Medical College (China). All surgical procedures were performed under intraperitoneal 4% chloral hydrate anesthesia, and the dose of chloral hydrate used for anesthesia of mice was 10 mL drug per kg animal body weight. The mouse UUO procedure was produced by ligation of the left ureter. Adeno-associated virus encoding miR-302 mimic or negative control (1.0×10^12^ vg) was injected via the tail vein after UUO surgery. Mice were sacrificed by cervical dislocation 7 days after left ureteral obstruction. The kidneys were collected for further evaluation.

### Cell culture and transfection

HK-2 cells were cultured in DMEM/F12 (HyClone; GE Healthcare, USA) medium supplemented with 10% fetal bovine serum (Gibco; Thermo Fisher Scientific, Inc., USA), 100 IU/mL streptomycin, and 100 IU/mL penicillin (Sigma Aldrich, USA) at 37°C and 5% CO_2_. For cell transfection, miR-302b mimics, miR-302b inhibitor, or corresponding negative control were diluted with OptiMEM I medium (Thermo Fisher Scientific, USA) and subsequently transfected into HK-2 cells using Lipofectamine 2000 (Invitrogen, USA). TGF-βR2 human cDNA ORF clone was purchased from KeyGen Biotec (China) and purified as previously described (12). HK-2 cells were transfected with TGF-βR2 plasmid or an empty vector by Lipofectamine 2000.

### Quantitative real-time PCR for miRNA and mRNA

Total RNA was extracted from kidney tissues or cells with TRIzol Reagent (Invitrogen), following to the manufacturer's instructions as previously described (12). Total RNA with the OD260/OD280 ratio between 1.7 and 2.1 was used for subsequent experiments. The first strand of cDNA was synthesized using a First-strand cDNA synthesis kit (Invitrogen). Quantitative real-time PCR was carried out subsequently to detect relative expression levels of miRNA and mRNA using the Quanti-Tect SYBR Green PCR mixture on an ABI 7500 fast real-time PCR system (Applied Biosystems, USA). The expression levels of U6 and β-actin were utilized for normalization of miRNAs and mRNA, respectively. The reaction conditions were: 2 min at 95°C, 25 s at 96°C, and 30 s at 63°C for 35 cycles. Relative expression levels of miR-302b and mRNAs were calculated by the 2^-ΔΔCT^ method. Primers for miR-302b and U6 were purchased from Ruibo (China). The primers for detection of mRNAs were as follows: collagen I (human), 5′-TGACGAGACCAAGAACTGCC-3′ (forward) and 5′-GCACCATCATTTCCACGAGC-3′ (reverse); α-SMA (human), 5′-CCCGGGACTAAGACGGGAAT-3′ (forward) and 5′-CCATCACCCCCTGATGTCTG-3′ (reverse); E-Cadherin (human), 5′-GCTGGACCGAGAGAGTTTCC-3′ (forward) and 5′-CAAAATCCAAGCCCGTGGTG-3′ (reverse); GAPDH (human), 5′-AATGGGCAGCCGTTAGGAAA-3′ (forward) and 5′-GCGCCCAATACGACCAAATC-3′ (reverse); Collagen I (mouse), 5′-GAGAGGTGAACAAGGTCCCG-3′ (forward) and 5′-AAACCTCTCTCGCCTCTTGC-3′ (reverse); α-SMA (mouse), 5′-CCTTCGTGACTACTGCCGAG-3′ (forward) and 5′-GTCAGCAATGCCTGGGTACAT-3′ (reverse); E-Cadherin (mouse), 5′-CCCCGAAAATGAAAAGGGCG-3′ (forward) and 5′-TGACGATGGTGTAGGCGATG-3′ (reverse); GAPDH (mouse), 5′-GACCTCATGGCCTACATGGC-3′ (forward) and 5′-CCGCATTAAAACCAAGGAGAGG-3′ (reverse).

### Histopathological analysis

Renal tissues from the four groups were fixed in 10% neutral buffered formalin, paraffin-embedded, and cut into 4-μm thick sections. The sections were then stained with Masson's trichrome staining (Solarbio, China). Tubulointerstitial fibrosis was assessed based on Masson's trichrome staining according to a previous study (13).

### Western blot analysis

The mouse renal tissues or HK-2 cells were lysed by RIPA lysis buffer (Beyotime, China) with 1% protease inhibitor cocktail (Roche, USA). Protein concentration was measured using a bicinchoninic acid kit (Thermo Scientific). After electrophoresis of samples on 6∼12% sodium dodecyl sulfate polyacrylamide gel, proteins were transferred onto a nitrocellulose membrane (Bio-Rad, USA), which were blocked in 5% skimmed dry milk (Bio-Rad). Then, membranes were incubated with primary antibodies against E-cadherin (cat. No. Ab76319; 1:1,000), α-SMA (cat. No. Ab32575; 1:1,000), TGF-βR2 (cat. No. Ab186838; 1:500), Smad2 (cat. No. Ab33875; 1:500), Smad3 (cat. No. Ab40854; 1:500), p-Smad2 (cat. No. Ab184557; 1:1,000), p-Smad3 (cat. No. Ab193297; 1:1,000), and GAPDH (cat. No. Ab181602; 1:1,000), all from Abcam (USA) overnight at 4°C followed by the appropriate horseradish peroxidase (HRP)-conjugated secondary antibodies for 1 h at room temperature. The bands were visualized using the ECL Western blotting detection kit (Bio-Rad) and analyzed with ImageJ software (Version 1.8.0, National Institutes of Health, USA).

### Luciferase reporter gene assay

The luciferase reporter gene was detected using a Dual-Luciferase Reporter Assay System (Promega, USA). The 3′-UTR seed sequence containing TGF-βR2 binding with miR-302b and mutated sequences were synthesized and cloned into the pmirGLO-REPORT Luciferase Vector (Promega). HK-2 cells were transfected with 200 ng miR-302b mimics or miR-302b inhibitors, subsequently co-transfected with 40 ng of the wild-type or mutant reporter vector using Lipofectamine 2000 (Invitrogen). Luciferase assay was conducted on cell lysates from the cells 48 h after transfection using Dual-Luciferase Assay System (Promega) according to the manufacturer's protocol. pmirGLO report vector was utilized as a positive control.

### Statistical analysis

The results are reported as means±SE. Comparisons between groups were performed using unpaired two tailed Student's *t* test or ANOVA, as appropriate. All statistical analyses were conducted using SPSS 19.0 (IBM, USA). P values less than 0.05 were regarded as statistically significant.

## Results

### Expression of miR-302b in renal tissue of UUO mice and TGF-β1-treated HK-2 cells

**Figure 1:**
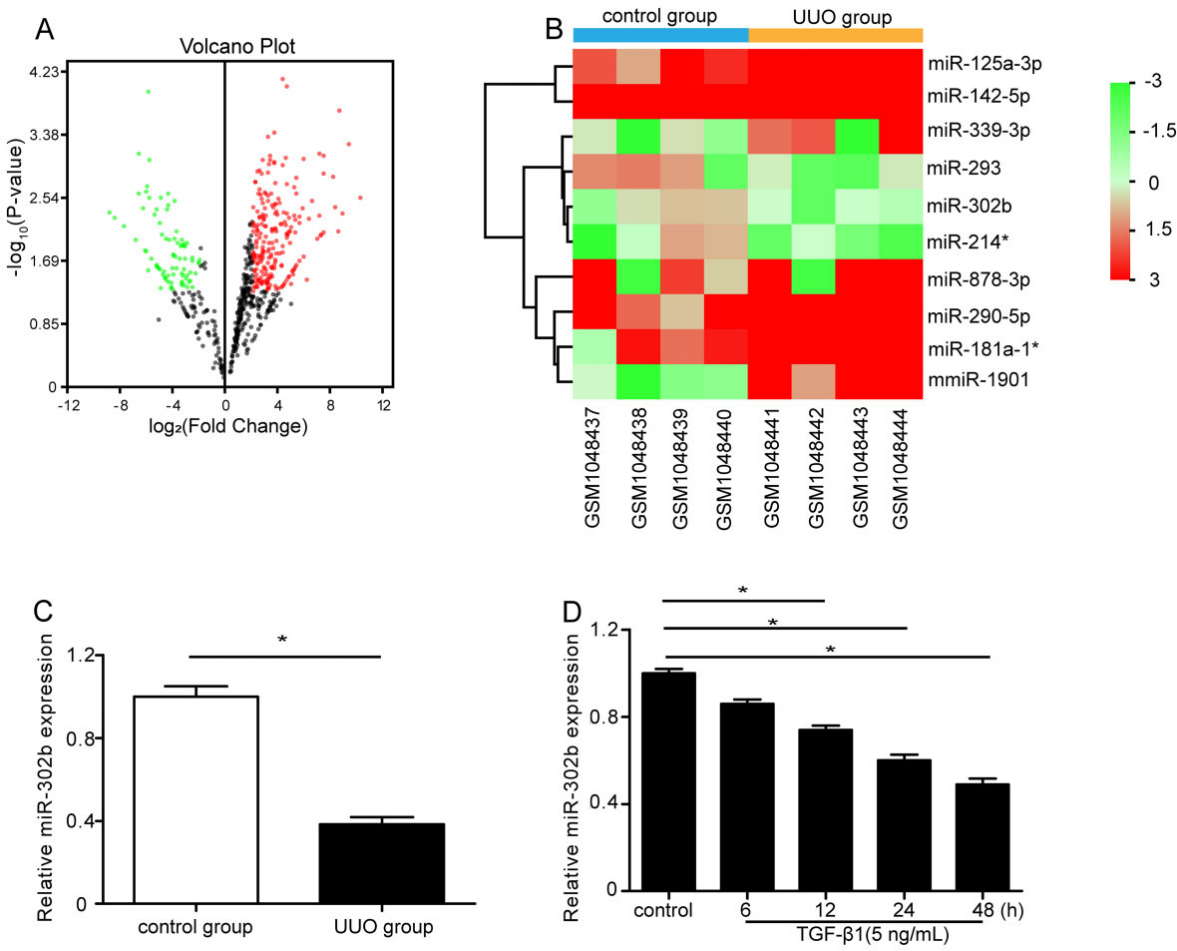
miR-302b was down-regulated in unilateral ureteral obstruction (UUO) mice and transforming growth factor β1 (TGF-β1)-treated HK-2 cells. **A**, Volcano plot of the differentially expressed miRNAs in gene expression profile datasets GSE42716. miRNAs that were not differentially expressed between the UUO group and the control group are represented as black dots. The red and green dots indicate up-regulated and down-regulated miRNAs, respectively. **B**, miR-302b displayed low expression in the UUO group based on microarray data (GSE42716) analysis. The abscissa and ordinate indicate sample number and differentially expressed miRNAs. miR-214* indicates miR-214-5p and miR-181a-1* indicates miR-181a-1-3p, according to miRbase website. Each square represents an expression value of sample. Red color indicates high expression and green color, low expression. **C**, miR-302b expression was measured by qRT-PCR (n=6). **D**, HK-2 cells were treated with saline or TGF-β1 (5 ng/mL) for 6, 12, 24, and 48 h. miR-302b expression was detected by qRT-PCR. Data are reported as means±SE (n=3). *P<0.05 (Student's *t*-test or ANOVA).

A total of 118 differentially expressed miRNAs including 71 upregulated and 47 downregulated were classified in the UUO group compared with the sham group. Differential expression miRNAs are reported as a volcano plot in Figure 1A. As shown in Figure 1B, miR-302b was significantly down-regulated in the UUO group. RT-PCR assay results showed that in the UUO group renal tissue, the expression of miR-302b was significantly down-regulated compared to the control group (Figure 1C). Furthermore, TGF-β1 treated HK-2 cells showed lower miR-302b expression (Figure 1D).

### Renal fibrosis and EMT progression were inhibited by miR-302b in UUO mice

**Figure 2:**
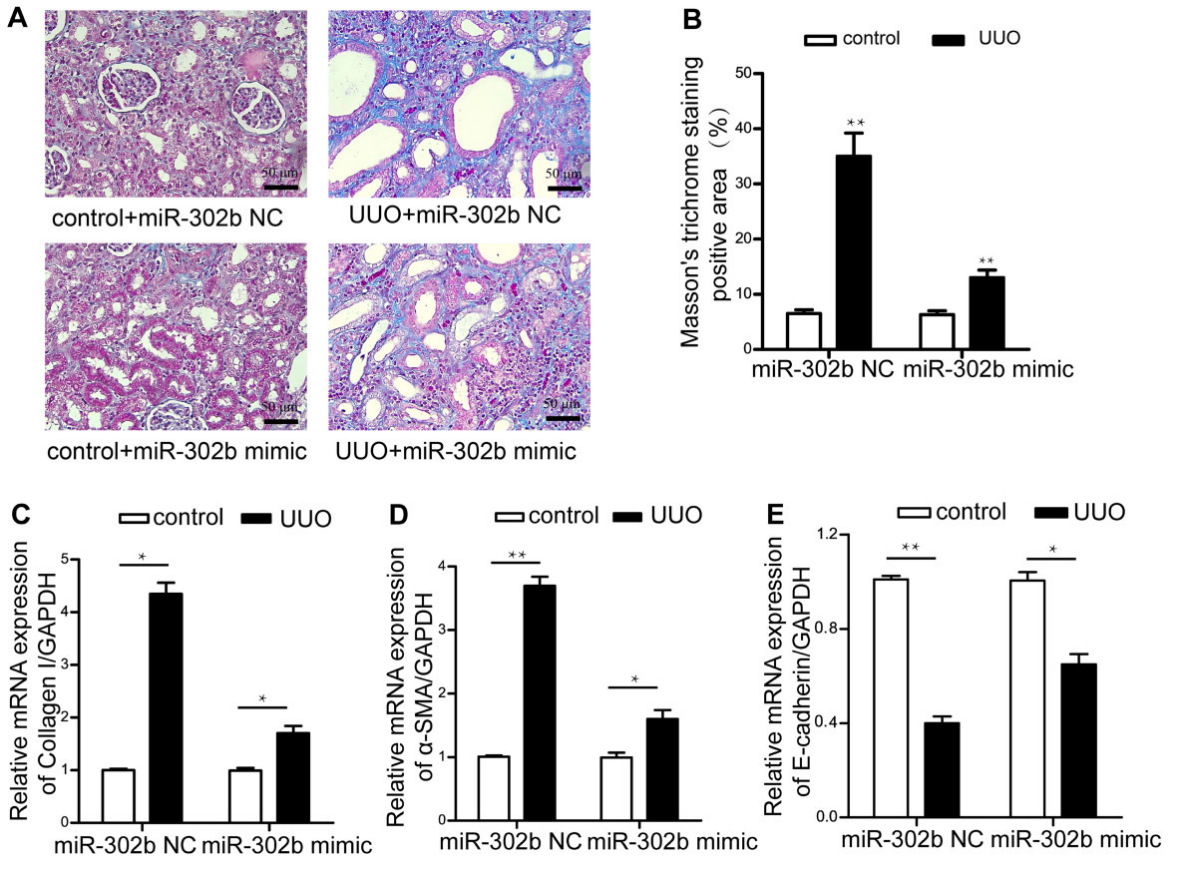
MiR-302b mimics reduced renal fibrosis and epithelial mesenchymal transition (EMT) progression. **A**, Representative Masson's trichrome staining of control and unilateral ureteral obstruction (UUO) mice treated with miR-302b mimics or negative control (NC) sequences. Magnification ×200; bars 50 μm. **B,** Quantitative analysis of fibrotic area in the different groups mentioned above. The percentage of Masson's trichrome staining positive area was calculated as follows: fibrotic area/total area of the field. Relative mRNA expression of Collagen I (**C**), α-smooth muscle actin (SMA) (**D**), and E-cadherin (**E**) in renal tissue were evaluated by qRT-PCR. Data are reported as means±SE (n=6). *P<0.05. **P<0.01 (Student's *t*-test).

As shown in Figure 2A and B, the percentage of Masson's trichrome staining positive area increased after UUO operation compared with the control group. However, miR-302b mimics decreased renal fibrosis detected by Masson's trichrome staining assay. Furthermore, expression of collagen I was down-regulated by miR-302b mimic shown by RT-PCR assay (Figure 2C). Previous studies have demonstrated a vital role of EMT progression in renal fibrosis. We confirmed that mRNA expression of α-SMA increased while E-cadherin decreased after UUO, and miR-302b mimics reversed those trends (Figure 2D and E).

### TGF-β1-induced EMT was arrested by miR-302b mimics in HK-2 cells

**Figure 3:**
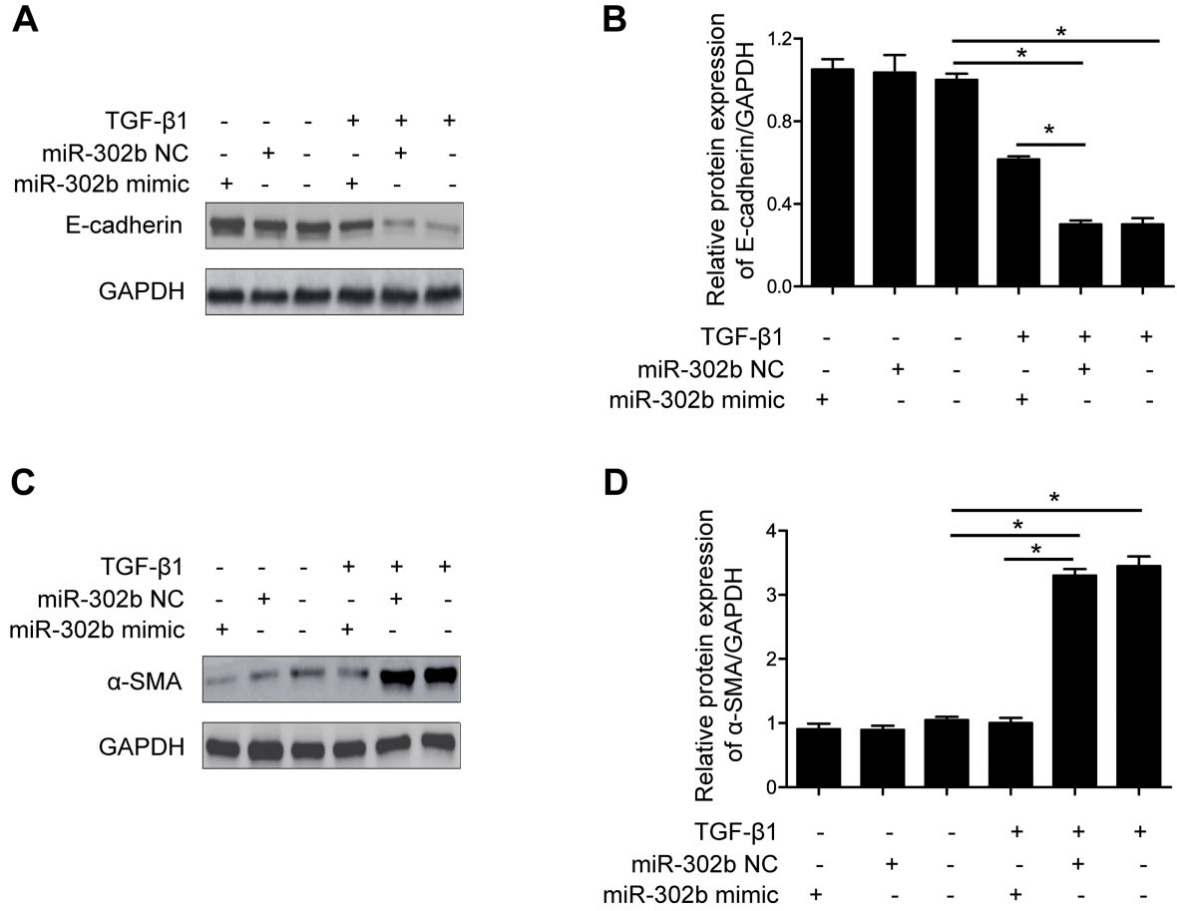
MiR-302b suppressed TGF-β1-induced epithelial mesenchymal transition (EMT) in HK-2 cells. Representative western blot images of E-cadherin (**A**) and α-SMA (**C**) in HK-2 cells treated with miR-302b negative control sequences (miR-302b NC, 20 nM) or mimics (miR-302b mimic, 20 nM ) with or without TGF-β1 (5 ng/mL) treatment for 48 h. Quantitative analysis of E-cadherin (**B**) and α-SMA (**D**) protein expression levels. Data are reported as means±SE (n=8). *P<0.05 (ANOVA).

MiR-302b mimic or negative control (NC) was transfected to HK-2 cells. Then cells were incubated with TGF-β1 (5 ng/mL) or corresponding vehicle (saline) for another 48 h. Compared with the vehicle group, TGF-β1-treated HK-2 cells showed lower E-cadherin (Figure 3A and B) and higher α-SMA expression (Figure 3C and D). However, in TGF-β1-treated cells, expression of E-cadherin was up-regulated (Figure 3A and B) and α-SMA expression was down-regulated in the miR-302b mimics-treated group compared to the NC group (Figure 3C and D).

### MiR-302b targeted TGF-βR2 directly

**Figure 4:**
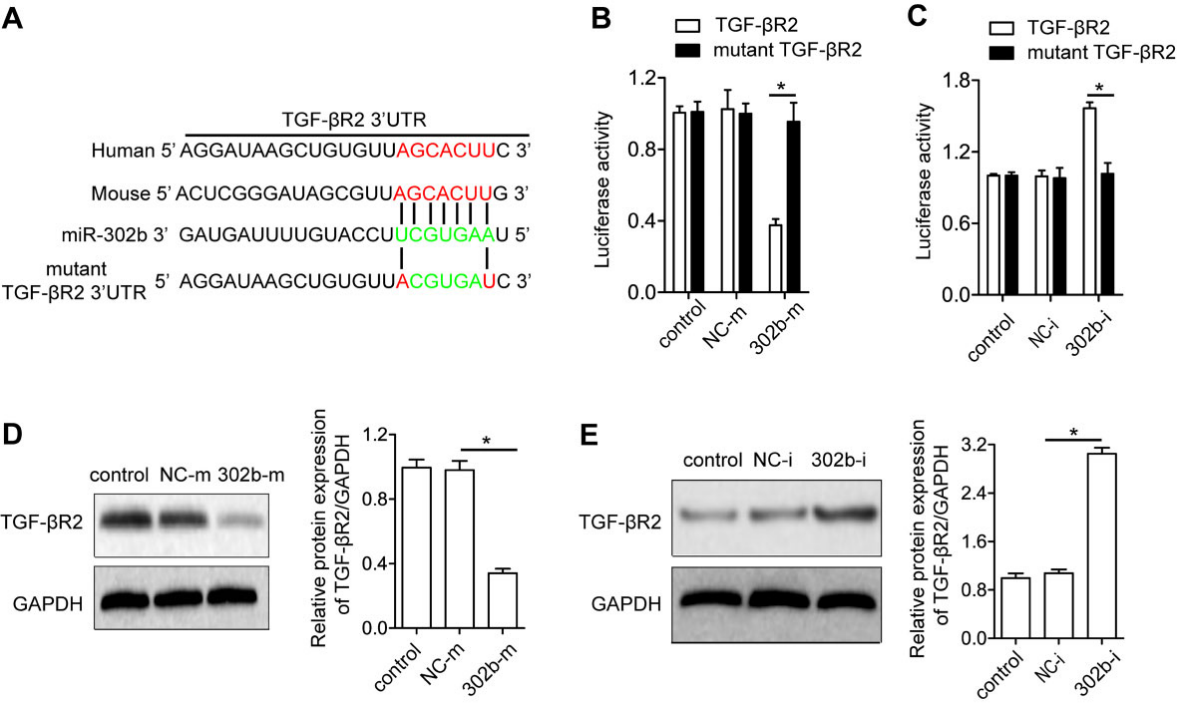
Identification of transforming growth factor (TGF)-βR2 as a target gene of miR-302b. **A**, Diagram of TGF-βR2 3′-UTR as a putative target for miR-302b in human and mouse. The seed-recognizing sites are indicated in red and mutated sequences are shown in green. Dual luciferase activity assay was performed in HK2 cells by co-transfection of luciferase reporter containing human wild type or mutant TGF-βR2 3′-UTR and miR-302b mimic (20 nM) (**B**), miR-302b inhibitor (20 nM) (**C**), or respective control sequences. miR-302b mimic decreased TGF-βR2 protein expression (**D**), and miR-302b inhibitor increased TGF-βR2 protein expression (**E**). Data are reported as means±SE (n=8). *P<0.05 (Student's *t*-test or ANOVA). NC-m: miR-302b mimic negative control, 302b-m: miR-302b mimic, NC-i: miR-302b inhibitor negative control, 302b-i: miR-302b inhibitor.

To explore underlying mechanisms of miR-302b regulating renal fibrosis, a computational mRNA target online analysis (http://www.targetscan.org) was conducted to predict potential target genes of miR-302b. Interestingly, miR-302b might directly target TGF-βR2 by binding to its 3′-UTR in mouse and human (Figure 4A). To further verify this prediction, firefly luciferase reporter constructs containing a wild-type or mutant 3′-UTR of TGF-βR2 was generated and luciferases reporter analysis was performed. miR-302 mimic significantly inhibited, while miR-302b inhibitor increased, the luciferase activity of TGF-βR2 3′-UTR. However, both miR-302b mimic and miR-302b inhibitor had no effect on the luciferase activity of luciferases reporter incorporating TGF-βR2 3′-UTR with the mutant miR-302b binding sites (Figure 4B and C). Consistent with the observation in TGF-βR2 3′-UTR reporter luciferase, miR-302b mimics significantly reduced TGF-βR2 protein expression while miR-302b inhibitor increased TGF-βR2 protein expression (Figure 4D and E). To sum up, TGF-βR2 was a target gene of miR-302b.

### MiR-302b mimics alleviated renal fibrosis and EMT by blocking TGF-β1/Smad signaling pathway *in vivo* and *in vitro*

**Figure 5:**
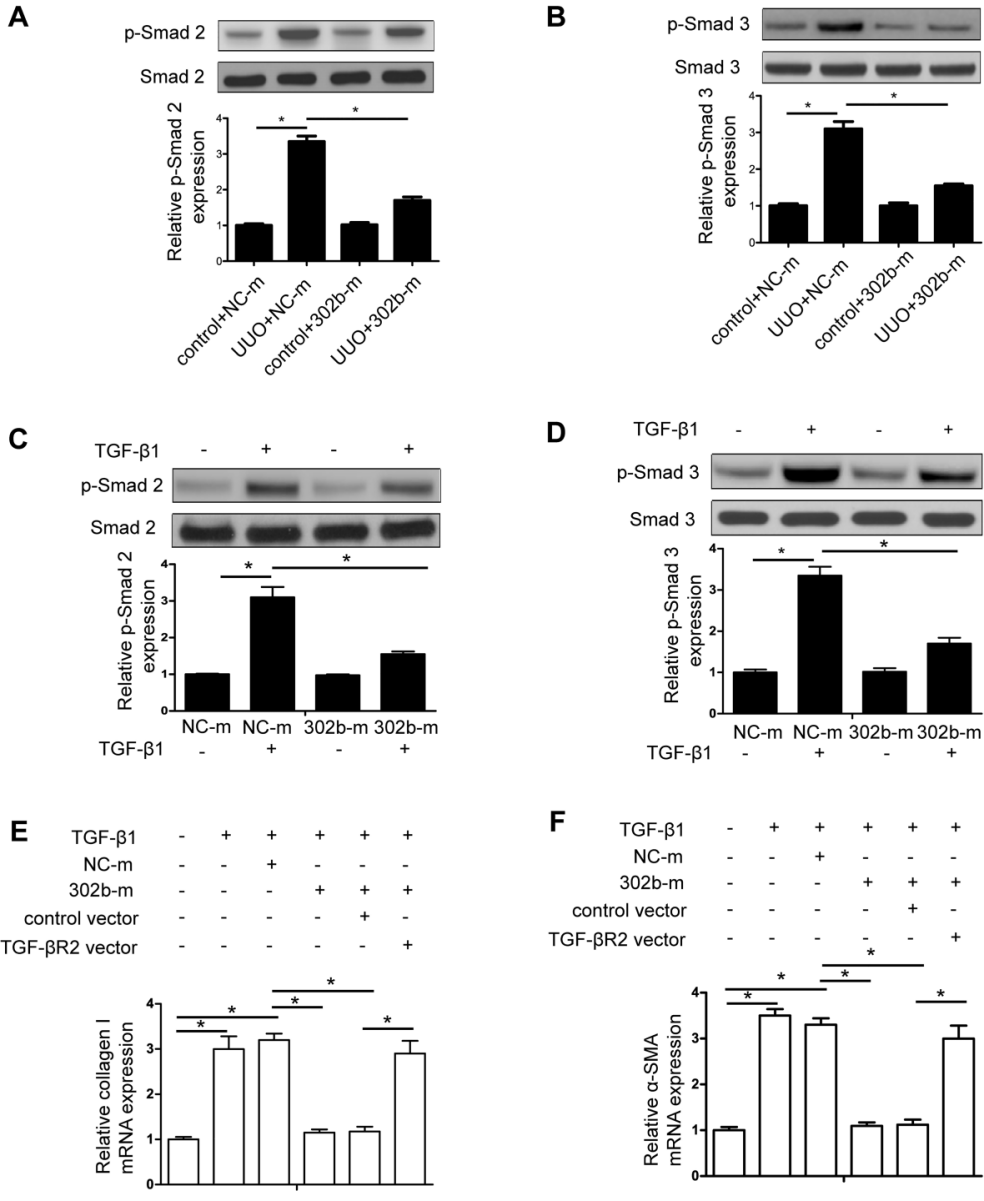
MiR-302b alleviated renal fibrosis and epithelial mesenchymal transition by inhibiting transforming growth factor (TGF)-β signaling pathway activation. Western blot analysis of Smad2 and p-Smad2 (**A**) and of Smad3 and p-Smad3 (**B**) expression in unilateral ureteral obstruction and control group mice treated by miR-302b mimic (20 nM) or negative control sequences (20 nM). HK-2 cells were transfected by miR-302b mimic or negative control sequences for 48 h, then treated with TGF-β1 (5 ng/mL) or saline for another 24 h. Expression of Smad2 and p-Smad2 (**C**) and of Smad3 and p-Smad3 (**D**) were detected by Western blot assay. qRT-PCR analysis of collagen I (**E**) and α-SMA (**F**) expression in HK-2 cells cotransfected with miR-302b mimic and TGF-βR2 vector for 48 h and then incubated with TGF-β1 or saline for another 24 h. Data are reported as means±SE (n=8). *P<0.05 (Student's *t*-test or ANOVA). NC-m: miR-302b mimic negative control, 302b-m: miR-302b mimic.

As shown in Figure 5A, in control mice, no difference in TGF-βR2 mRNA expression was observed in miR-302b-treated mice or NC. However, in UUO mice, miR-302b mimics significantly reduced TGF-βR2 expression in mRNA level. Moreover, whether treated with miR-302b mimics or NC, there were no obvious differences of Smad-2 and Smad-3 phosphorylation in the control group mice, while miR-302b mimics decreased Smad-2 and Smad-3 phosphorylation in UUO mice (Figure 5B and C). In accordance with *in vivo* findings, miR-302 mimics inhibited TGF-β1-induced TGF-βR2 expression (Figure 5D), and Smad-2 and Smad-3 phosphorylation (Figure 5E and F) in HK-2 cells. Furthermore, TGF-β1-induced collagen I and α-SMA accumulation were abolished by miR-302 mimics (Figure 5D).

## Discussion

Progressive renal fibrosis is an end-stage of obstructive nephropathy and results in renal insufficiency (1). Until now, no effective therapeutic strategy exists for renal fibrosis (14). Therefore, it is imperative to explore the exact cellular and molecular mechanisms of this pathologic process.

MicroRNAs are a group of noncoding small RNAs approximately 22 nucleotides in length. By binding to 3′-untranslated regions of mRNA, miRNAs can suppress translation of target genes (15). miR-302b has been proven to be involved in many important pathologic processes including suppressing lung cancer cell proliferation (16), inhibiting osteosarcoma cell invasion (17), and accelerating the skin aging process (18). Recently, more and more studies have focused on the interaction between miR-302 family members and renal cell fibrosis. The expression of miR-302d is increased in UUO mouse, and miR-302d inhibits epithelial mesenchymal transition progression of renal HKC8 epithelial cells induced by TGF-β (19). Microvesicles released from mesenchymal stem cells incubated in erythropoietin restore kidney damage caused by UUO *in vivo* and increased miR-302b expression. In addition, these microvesicles inhibit HK-2 cell fibrosis induced by TGF-β1. This study indicates that miR-302b may contribute to renal fibrosis prevention (20). A recent study demonstrates that overexpression of miR-302 facilitates human mesangial cell plasticity characterized by increased expression of Snail (21). Mesangial cells can participate in the progression of renal glomerular fibrosis by secreting inflammatory cytokines, adhesion molecules, and chemokines (22). Moreover, Snail-induced EMT process contributes to tubulointerstitial fibrosis in diabetic nephropathy (23). In addition, miR-302c is proven to prevent mesothelial cells fibrosis and mesothelial‐mesenchymal transition induced by TGF‐β1 through targeting connective tissue growth factor during peritoneal dialysis (24). In summary, these studies suggest that miR-302 family members may function as protective factors in the pathological process of cell fibrosis, especially renal cells.

In our present study, microarray analysis revealed that kidney miR-302b was significantly decreased after UUO in mice. According to this finding, we established a mouse UUO model. In accordance with the results of microarray analysis, the expression of miR-302b was down-regulated in the UUO group compared with the control group detected by qRT-PCR. TGF-β1-treated human kidney tubular cells (HK-2 cells) is a classical *in vitro* model of renal cell fibrosis (25,26). We found a significant decrease in expression of miR-302b in TGF-β1 treated HK-2 cells, which was similar to the *in vivo* results. These initial results suggested miR-302b may participate in renal fibrosis progression both *in vivo* and *in vitro*. To verify this hypothesis, miR-302b mimics or negative control sequences were injected in both UUO and sham-operation mice. Results demonstrated that miR-302b attenuated renal fibrosis detected by Masson's trichrome staining. Meanwhile, miR-302b up-regulation suppressed type I collagen and α-SMA expression and increased E-cadherin expression. Previous studies have demonstrated that collagen I, an important component of interstitial extracellular matrix, is up-regulated during renal fibrosis (27,28). Increased α-SMA expression and loss of E-cadherin indicates renal cells undergo EMT, which is an important part of renal fibrosis (29,30). EMT involving injured epithelial cells performs vital roles in the pathological process of renal fibrosis. Tubular epithelial cells undergoing EMT can obtain a mesenchymal phenotype, which is believed to be a source of myofibroblasts/fibroblasts (31,32). Myofibroblasts have been demonstrated to be an important contributor of collagen I synthesis during renal fibrosis (33). Results from animal experiments show that miR-302b alleviates renal fibrosis through inhibiting unusual accumulation of extracellular matrix and EMT of renal cells. Whether miR-302b has effects on EMT of renal tubular epithelial cells is still unknown. TGF-β1 is considered to be a key factor to induce EMT of renal tubular epithelial cells during renal fibrosis progression (9). In line with results obtained from *in vivo* experiments, HK-2 cells treated with TGF-β1 displayed higher α-SMA expression and lower E-cadherin expression while miR-302b reversed this change suggesting that miR-302b could inhibit TGF-β1-induced EMT of renal tubular epithelial cells.

To further explore the underlying mechanisms, bioinformatics analysis was conducted. TargetScan database indicated that TGF-βR2 has a target site for miR-302b. TGF-β1 contacting with TGF-βR2 results in recruitment and phosphorylation of TGF-βR1. Then, kinase domains within the receptors are activated (34). In order to validate this prediction, dual-luciferase activity assay and western blot assay were performed. Based on our observations, TGF-βR2 was proven to be a functional target of miR-302b in HK-2 cells. Furthermore, previous studies have demonstrated that targeting TGF-βR2 can inhibit interstitial renal fibrosis in UUO mice via Smad-dependent mechanism (13,35). Activation of Smad2 and Smad3 potentiates several profibrotic gene expressions containing collagens (36), integrins (37), connective tissue growth factor (38), and matrix metalloproteinases (39,40). Therefore, we hypothesized that miR-302b inhibited renal fibrosis by targeting TGF-βR2 via suppressing TGF-β/Smad pathway. Our following *in vivo* and *in vitro* experiments supported this hypothesis. Increasing phosphorylation of Smad2 and Smad3 was observed in kidney tissue after UUO in mice, whereas miR-302b overexpression mitigated this change. In agreement with the *in vivo* findings, TGF-β1-treated HK-2 cells exhibited high phosphorylation of Smad2 and Smad3. However, miR-302b up-regulation decreased Smad2 and Smad3 phosphorylation. These findings demonstrated that miR-302b suppressed TGF/Smad pathway activation. Moreover, for the sake of exploring a definite relationship between miR-302b and TGF-βR2 in renal fibrosis regulation, we promoted TGF-βR2 expression by transfecting designed TGF-βR2 plasmid into HK-2 cells and observed restoration of collagen I and α-SMA expression compared to miR-302b transfection alone. These results indicated that miR-302b inhibited renal cell fibrosis mainly through targeting TGF-βR2.

In summary, our present study demonstrated for the first time that overexpression of miR-302b attenuated renal fibrosis through suppressing TGF-β/Smad-dependent pathway by targeting TGF-βR2. These results indicate a possible therapeutic strategy for the prevention of fibrotic kidney diseases.
